# Intracellular accumulation of tau inhibits autophagosome formation by activating TIA1-amino acid-mTORC1 signaling

**DOI:** 10.1186/s40779-022-00396-x

**Published:** 2022-07-07

**Authors:** Meng-Zhu Li, En-Jie Liu, Qiu-Zhi Zhou, Shi-Hong Li, Shi-Jie Liu, Hai-Tao Yu, Qi-Hang Pan, Fei Sun, Ting He, Wei-Jin Wang, Dan Ke, Yu-Qi Feng, Jun Li, Jian-Zhi Wang

**Affiliations:** 1 Department of Pathophysiology, School of Basic Medicine, Key Laboratory of Education Ministry of China/Hubei Province for Neurological Disorders, Tongji Medical College, Huazhong University of Science and Technology, Wuhan, 430030 China; 2 Department of Neurosurgery, the Central Hospital of Wuhan, Tongji Medical College, Huazhong University of Science and Technology, Wuhan, 430014 China; 3 Department of Pathology, The First Affiliated Hospital of Zhengzhou University, Zhengzhou, 450052 China; 4 Department of Chemistry, Wuhan University, Wuhan, 430072 China; 5 Co-Innovation Center of Neuroregeneration, Nantong University, Nantong, 226000 Jiangsu China

**Keywords:** Tau, Autophagy, Amino acid pathway, Mammalian target of rapamycin kinase complex 1 (mTORC1), T cell intracellular antigen 1 (TIA1)

## Abstract

**Background:**

Autophagy dysfunction plays a crucial role in tau accumulation and neurodegeneration in Alzheimer’s disease (AD). This study aimed to investigate whether and how the accumulating tau may in turn affect autophagy.

**Methods:**

The primary hippocampal neurons, N2a and HEK293T cells with tau overexpression were respectively starved and treated with vinblastine to study the effects of tau on the initiating steps of autophagy, which was analysed by Student's two-tailed *t*-test. The rapamycin and concanamycin A were employed to inhibit the mammalian target of rapamycin kinase complex 1 (mTORC1) activity and the vacuolar H^+^-ATPase (v-ATPase) activity, respectively, which were analysed by One‐way ANOVA with post hoc tests. The Western blotting, co-immunoprecipitation and immunofluorescence staining were conducted to gain insight into the mechanisms underlying the tau effects of mTORC1 signaling alterations, as analysed by Student's two-tailed *t*-test or One‐way ANOVA with post hoc tests. The autophagosome formation was detected by immunofluorescence staining and transmission electron microscopy. The amino acids (AA) levels were detected by high performance liquid chromatography (HPLC).

**Results:**

We observed that overexpressing human full-length wild-type tau to mimic AD-like tau accumulation induced autophagy deficits. Further studies revealed that the increased tau could bind to the prion-related domain of T cell intracellular antigen 1 (PRD-TIA1) and this association significantly increased the intercellular level of amino acids (Leucine, *P* = 0.0038; Glutamic acid, *P* = 0.0348; Alanine, *P* = 0.0037; Glycine, *P* = 0.0104), with concordant upregulation of mTORC1 activity [phosphorylated eukaryotic translation initiation factor 4E-binding protein 1 (p-4EBP1), *P* < 0.0001; phosphorylated 70 kDa ribosomal protein S6 kinase 1 (p-p70S6K1), *P* = 0.0001, phosphorylated unc-51-like autophagy-activating kinase 1 (p-ULK1), *P* = 0.0015] and inhibition of autophagosome formation [microtubule-associated protein light chain 3 II (LC3 II), *P* = 0.0073; LC3 puncta, *P* < 0.0001]. As expected, this tau-induced deficit of autophagosome formation in turn aggravated tau accumulation. Importantly, we also found that blocking TIA1 and tau interaction by overexpressing PRD-TIA1, downregulating the endogenous TIA1 expression by shRNA, or downregulating tau protein level by a small proteolysis targeting chimera (PROTAC) could remarkably attenuate tau-induced autophagy impairment.

**Conclusions:**

Our findings reveal that AD-like tau accumulation inhibits autophagosome formation and induces autophagy deficits by activating the TIA1/amino acid/mTORC1 pathway, and thus this work reveals new insight into tau-associated neurodegeneration and provides evidence supporting the use of new therapeutic targets for AD treatment and that of related tauopathies.

## Background

Alzheimer’s disease (AD) is the most common cause of senile dementia and is characterized pathologically by the massive formation of intercellular neurofibrillary tangles (NFTs) and extracellular plaques, which comprise hyperphosphorylated tau (p-tau) and β-amyloid (Aβ), respectively [1]. Tau is a microtubule-associated protein with the main function of promoting microtubule assembly and maintaining the stability of the microtubules. As seen in AD and related neurodegenerative disorders, abnormal hyperphosphorylation converts normal tau from a microtubule assembly promoting to a microtubule assembly disrupting protein [2–4]. In the brain tissue and cerebrospinal fluid of AD patients, the level of tau protein is approximately 4–8 times that of normal people [5, 6], and the accumulation of hyperphosphorylated tau is positively correlated with the cognitive decline demonstrated by clinicopathologic investigations [7, 8]. Both in vitro and in vivo experimental studies also show that tau accumulation in different brain regions impairs synaptic/circuit functions and causes learning and memory deficits [9–12].

Tau accumulation could be induced by increased protein synthesis or impaired proteolysis, and studies suggest that the latter is the major cause of tau accumulation in AD patients. Autophagy is one of the major cellular mechanisms for the renewal of cytoplasmic components and mainly occurs during nutritional crisis or starvation [13]. In the brains of accelerated aging mice, an accumulation of ubiquitinated proteins and a reduction in autophagy activity were detected at 12 months of age [14]. The autophagy-related genes (*Atg5*, *Atg7*) in the brains of the elderly individuals were significantly downregulated [15], and the mRNA and protein levels of the autophagy-related protein beclin-1 in the brains of early AD patients were reduced [16]. Suppression of basal autophagy by downregulating the autophagy protein 5 (ATG5) caused neurodegeneration in mice [17]. In addition, lysosome abnormalities in the brains of transgenic mice overexpressing mutated 4R-tau (G272V, P301L and R406W) were detected by electron microscopy [18]. These observations strongly suggest that autophagy deficits could contribute to tau accumulation in AD. However, it is unclear whether the accumulated tau in turn contributes to the autophagy deficit during the chronic progression of AD.

Indeed, we found in a recent study that AD-like tau accumulation could disrupt autophagosome-lysosome fusion by deregulating the Acidic nuclear phosphoprotein 32 family member A-Inhibitor of histone acetyl transferase-Increased sodium tolerance 1-Endosomal sorting complex required for transport III (ANP32A-INHAT-IST1-ESCRT-III) pathway, and this study preliminarily revealed a vicious cycle of tau accumulation and autophagy deficit in chronic AD neurodegeneration [19]. During the above study, we also observed a significantly reduced level of protein synthesis and reduced the microtubule-associated protein light chain 3 II (LC3 II) level induced by tau, which could not be explained by the autophagosome-lysosome fusion deficit. It is well known that macroautophagy is roughly divided into four consecutive steps, including autophagy initiation, autophagosome formation, membrane expansion, and autophagy maturation [20]. Autophagosome formation involves the mammalian target of rapamycin (mTOR), a conserved serine/threonine protein kinase that mainly forms two types of complexes, the mammalian target of rapamycin kinase complex 1 (mTORC1) and the mammalian target of rapamycin kinase complex 2 (mTORC2). mTORC1 responds to nutritional signals, such as amino acids (AAs), insulin and growth factors, from inside and outside the cell to maintain normal cell survival by regulating intracellular RNA transcription, protein synthesis, and autophagy. mTORC1 inhibits the formation of the Unc-51-like autophagy-activating kinase 1-Autophagy protein 13-FAK family kinase-interacting protein of 200 kDa (ULK1-ATG13-FIP200) autophagy-related complexes by phosphorylating ULK1/2 and ATG13, thereby inhibiting the initiation of autophagy process [21]. Activation of mTORC1 has been observed in AD [22, 23], and downregulating mTORC1 ameliorated AD-like pathologies and cognitive deficits [24]. Meanwhile, studies also show that rapamycin can prolong the lifespan of mice [25, 26].

Currently, the effect of tau accumulation on the initiating steps of autophagy has not been reported. Therefore, we investigated whether and how tau accumulation affects autophagosome formation, the initiating part of autophagy, by using inhibitors of autophagosome-lysosome fusion and measurements of the mTORC1 pathway. 

## Methods

In the present study, we used Western blotting, immunofluorescence, co-immunoprecipitation, high performance liquid chromatography (HPLC) in cells, rat primary neurons and transgenic mice to reveal the role and the molecular mechanisms underlying tau-induced dysfunction of autophagosome formation. Mice were divided into the human tau (hTau) overexpression group and the endogenous mouse tau knockout (tau^−/−^) group to reveal the in vivo effect of tau accumulation on the mTORC1 pathway (*n* = 9 for each group). To explore whether down-regulating tau protein in vivo could attenuate the autophagy dysfunction induced by tau, mice were treated with vehicle or C004019 (C4), a novel small-molecule proteolysis targeting chimera (PROTAC), and divided into 4 groups, including wild type (WT) group, 3 × Tg AD group, WT plus C4 group and 3 × Tg AD plus C4 group (*n* = 9 for each group). All animal experiments were approved by the Ethics Committee of Tongji Medical College (IACUC Number: 2735).

### Animals and drug administration

Human tau transgenic mice, the endogenous mouse tau knockout (tau^−/−^) transgenic mice and 3 × Tg-AD mice harboring knock-in of the Swedish double mutation of amyloid precursor protein (APP), a presenilin 1 mutation (Psen1tm1Mpm), and a frontotemporal dementia tau mutation (tauP301L) were kind gifts of Dr. Xi-Fei Yang (Laboratory of Modern Toxicology of Shenzhen, Shenzhen Centers for Disease Control and Prevention, Nanshan District, Shenzhen, China).

For the tau overexpressing model, mice were divided into two groups: the tau-group (*n* = 9, three independent experiments) and the hTau group (*n* = 9, three independent experiments). For subcutaneous injection of 3 × Tg-AD mice, C004019 (C4), a novel small-molecule proteolysis targeting chimera (PROTAC), was diluted with 20% 2-Hydroxylpropyl-beta-cyclodextrin (HP-β-CD) and injected under the skin on the back neck to reach a final concentration of 3 mg/kg (every 6 d for a total of 5 times in one month) [27]. Mice were treated with vehicle or C004019 and divided into four groups: the wild type (WT) group (*n* = 9, three independent experiments), 3 × Tg AD group (*n* = 9, three independent experiments), WT plus C4 group (*n* = 9, three independent experiments) and 3 × Tg AD plus C4 group (*n* = 9, three independent experiments).

All mice were kept at (24 ± 2) °C with access to food and water under a 12 h light/dark cycle. All surgical and irradiation procedures were performed under anesthesia, and animal suffering was minimized as much as possible. Pentobarbital sodium anesthetic (50 mg/kg) was intraperitoneally injected to anesthetize mice, and fresh brain hippocampus tissues were removed and stored at − 80 °C.

### Cell culture

HEK293T cell lines were purchased from the Chinese Academy of Sciences Cell Bank (Shanghai, China) and cultured in DMEM/HIGH medium containing 10% fetal bovine serum (FBS), while the N2a cell lines were purchased from the Chinese Academy of Sciences Cell Bank (Shanghai, China) and cultured in a 1:1 mixture of Dulbecco’s modified Eagle’s medium and OPTI-MEM supplemented with 10% fetal bovine serum (FBS), in a humidified atmosphere of 5% CO_2_ at 37 °C. The cells were plated into 12-well plates, 6-well plates, or fluorescent slides for plasmid transfection and/or drug treatment.

### Primary neuron culture

For primary hippocampal neuron cultures, 18 d embryonic rat hippocampal neurons were plated at 30,000–40,000 cells per well on 6-well plates coated with Poly-D-Lysine/Laminin (Sigma-Aldrich, St. Louis, Missouri, USA) in neurobasal medium (ThermoFisher Scientific, Waltham, Massachusetts, USA) supplemented with 2% B27 (a defined yet complex mixture of antioxidant enzymes, proteins, vitamins, and fatty acids that are combined in optimized ratios to support neuronal survival in culture) /0.5 mmol/L glutamine/25 mmol/L glutamate. Half the culture medium was changed every 3 d with neurobasal medium supplemented with 2% B27 (ThermoFisher Scientific, Waltham, Massachusetts, USA) and 0.5 mmol/L GlutaMAX (ThermoFisher Scientific, Waltham, Massachusetts, USA). All cultures were kept at 37 °C in a humidified 5% CO_2_ containing atmosphere. On Day 7 of in vitro culture, the neurons were transfected with lentivirus Lenti-eGFP-hTau, Lenti-eGFP-C1, Lenti-eGFP-P2A-hTau or Lenti-eGFP-P2A-C1, which were packaged by OBiO (Shanghai, China). The conditioned culture medium was replaced 1/2 volume with fresh feeding media every 3 d for neuron maintenance until the neurons were ready to use for experiments on Day 14. Specificity of the primary neuron was confirmed by Immunofluorescence with NeuN, GFAP, and Iba1 antibodies, the Marker of neuron, astrocyte, and microglia respectively, and the percent of neuron was at least 95%.

### Western blotting

The treated cells, primary cultured neurons and hippocampus tissues were lysed with RIPA lysis buffer and then used for Western blotting. Proteins were separated by 8%, 10% or 12% SDS–polyacrylamide gel, electrophoresis and transferred to NC membrane, blocked with 5% nonfat milk or 3% BSA, which was dissolved in TBS-Tween-20 (50 mmol/L Tris HCl, 150 mmol/L NaCl, 0.2% Tween-20, pH 7.6) for 1 h and incubated with primary antibodies [anti-RAC serine/threonine protein kinase (AKT), anti-Phospho-AKT, anti-Phospho-70 kDa ribosomal protein S6 kinase 1 (p70S6K1), anti-Phospho-eukaryotic translation initiation factor 4E-binding protein 1 (4EBP1), anti-ULK1, anti-Phospho-ULK1 and anti-Phospho-AMP-activated protein kinase (AMPK) (Cell Signaling Technology, Danvers, Massachusetts, USA); anti-p70S6K1, anti-4EBP1, anti-ATG13, anti-GFP (Proteintech, Wuhan, Hubei, China); anti-Flag and anti-AMPK (Sigma-Aldrich, St. Louis, Missouri, USA); anti-LC3B, anti-β-actin and anti-Tau5 (Abcam, Cambridge UK); anti-T cell intracellular antigen 1 (TIA1) (Santa Cruz Biotechnology, Paso Robles, California, USA); anti-Puromycin (Merck-Millipore, Boston, Massachusetts, USA)] at 4 °C overnight. On the second day, the membranes were incubated with anti-rabbit or anti-mouse secondary antibodies at room temperature for 1 h and visualized using the Odyssey Infrared Imaging System (version 3.0, Licor Biosciences, Lincoln, NE, USA) and the ECL Imaging System (version 2017.11.14.0, Clinx Science Instruments Co., Ltd, Shanghai, China).

### Immunofluorescence

The hippocampal neurons infected with lentivirus of tau and cells transfected with plasmids of tau in slices were fixed with paraformaldehyde for 30 min, ruptured with 0.5% TritonX-100 in PBS, blocked with 5% BSA in PBS, and incubated with primary antibodies [anti-mTOR (Cell Signaling Technology, Danvers, Massachusetts, USA), anti-lysosome-associated membrane glycoprotein 1(LAMP1) (Abcam, Cambridge UK), anti-TIA1 (Santa Cruz Biotechnology, Paso Robles, California, USA)] for 24 h at 4 °C. On the second day, the slices were washed with 0.1% TritonX-100 in PBS 3 times and incubated with secondary antibodies for 1 h at room temperature. Then, the slices were washed with 0.1% TritonX-100 in PBS 3 times and incubated with Hoechst for 10 min. Finally, the slices were washed with 0.1% TritonX-100 in PBS 3 times and covered with 50% glycerin/PBS. Pictures were visualized by a Confocal Microscope Zeiss Carl LSM780 (version 8.0, Zeiss Carl LSM 780, Oberkochen, Baden-Württemberg, Germany).

### Quantification of the levels of DsRED-LC3-positive puncta

The quantification of the levels of DsRED-LC3-positive puncta was referred to the published literature [28]. HEK293T cells were plated on uncoated φ14-mm glass coverslips, transfected with plasmids for 42 h, and then exposed to starvation and 50 μmol/L vinblastine with or without rapamycin for 6 h. Cells were imaged on a Confocal Microscope Zeiss Carl LSM780 (version 8.0, Zeiss Carl LSM 780, Oberkochen, Baden-Württemberg, Germany) oil immersion 63 × objective. A minimum of three areas per coverslip were imaged. We counted the number of DsRED-LC3 puncta in individual cells in each group of samples, counting 22 – 42 cells per group, and performed at least 3 independent experiments.

### Co-immunoprecipitation (Co-IP)

Proteins were extracted on ice using RIPA buffer (Beyotime, Shanghai, China), which contained 20 mmol/L Tris–HCl, pH 7.5, 150 mmol/L NaCl, 1% Trixon X-100, 1% sodium deoxycholate, 1 mmol/L EDTA, 50 mmol/L N-ethylmaleimide, 1 mmol/L NaF, 1 mmol/L Na_3_VO_4_, and 1 μg/mL each of aprotinin, leupeptin, pepstatin, and 1 mmol/L phenylmethane sulfonyl fluoride, centrifuged at 12,000 × g for 5 min at 4 °C, and the suspension was rotated with primary antibodies [anti-ULK1 (Cell Signaling Technology, Danvers, Massachusetts, USA), anti-ATG13 and anti-GFP (Proteintech, Wuhan, Hubei, China), anti-Flag (Sigma-Aldrich, St. Louis, Missouri, USA)] at 4 °C overnight. Protein G agarose (Merck Millipore, Boston, Massachusetts, USA) was washed with PBS buffer and centrifuged at 6000×*g* for 30 s at 4 °C, and the supernatant was removed. Washed protein G was added to the extracts (1 μg/μL protein) and rotated at 4 °C for 6 h. After centrifugation at 12,000×*g* for 5 min at 4 °C, the agarose beads were collected, washed with precooled washing buffer (PBS, protease inhibitors, 0.5% Nonidet P-40, and 0.1% Triton X-100) 5 times, and centrifuged for 1 min at 12,000×*g* after each wash. The agarose beads were collected and resuspended in 2 × SDS-PAGE loading buffer, boiled for 5 min and centrifuged. The supernatant was analysed by Western blotting.

### High performance liquid chromatography (HPLC)

HEK293T cells were plated into 35 mm cell culture dishes. When the cell density reached 80%, the cells were transiently transfected with eGFP-tau or eGFP-C1 plasmids. After 42 h, the culture medium was changed to DMEM/High without FBS for another 6 h. Then, the cell supernatant was removed and the cells were washed with PBS buffer 3 times. Next, the extraction reagent was configured according to the volume ratio of methanol: acetonitrile: ddH2O (5:3:2). Then, 2 ml of extraction reagent was added to each plate, and quickly frozen at − 80 °C, thawed at 4 °C and shaken for 30 min. After filtering, high performance liquid chromatography was performed to detect the concentration of amino acids.

### Protein synthesis detection

The level of protein synthesis was detected by puromycin and Puromycin antibodies [29]. N2a cells were transfected with plasmids for 48 h, 10 µg/mL puromycin in prewarmed complete culture medium was added, and the cells were incubated for 10 min at 37 °C and 5% CO_2_, gently washed two times with prewarmed complete culture medium and incubated for 50 min at 37 °C and 5% CO_2_ with prewarmed complete culture medium. Cells were harvested, and protein synthesis was detected by Western blotting using an anti-puromycin antibody (12D10).

### Statistical analysis

All data conform to Normal distribution and are presented as the mean ± SEM. GraphPad Prism (version 8.0, GraphPad Software, San Diego, California, USA) was used for statistical analyses. The Student’s *t*-test was used for two-group comparisons. One‐way ANOVA with post hoc tests was conducted for multiple comparisons. *P* < 0.05 was considered statistically significant.

## Results

### Tau accumulation disrupts autophagosome formation leading to autophagy deficit

**Fig. 1:**
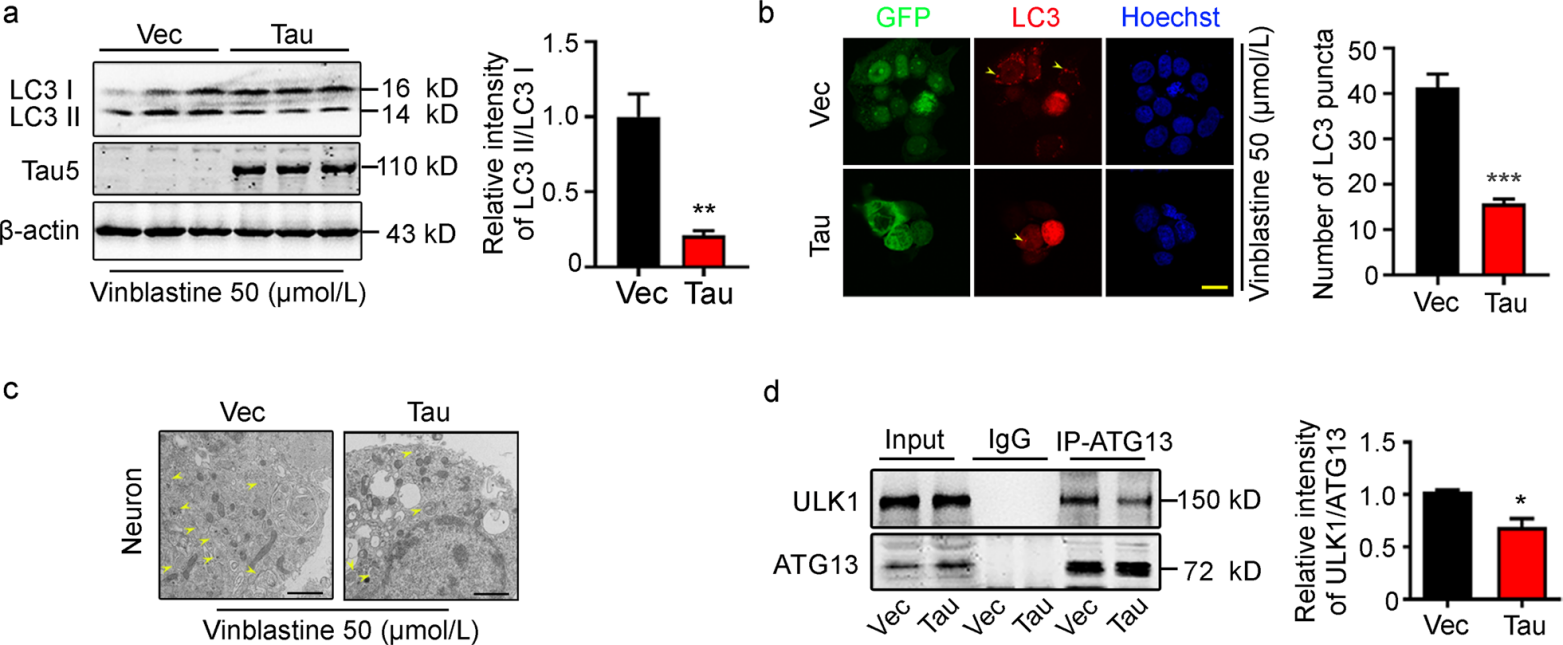
Overexpressing tau inhibits autophagy and disrupts autophagosome formation. **a** Overexpressing tau decreased LC3 II protein levels in HEK293T cells, as detected by Western blotting. The LC3 II level was normalized against LC3 I (*n* = 9). **b** Overexpressing tau decreased numbers of LC3 puncta in HEK293T cells, as measured by immunofluorescence staining (at least 30 cells were analyzed for each group, scale bar = 20 μm). The yellow arrow points to LC3 puncta. **c** Overexpressing tau decreased the autophagosome formation in primary hippocampal neurons, as measured by transmission electron microscopy, scale bar = 1 μm. The yellow arrow points to the autophagosome. **d** Overexpressing tau decreased autophagosome formation of HEK293T cells, as measured by coimmunoprecipitation of ULK1 and ATG13 (*n* = 6). ATG13 autophagy protein 13, GFP green fluorescent protein, IP immunoprecipitation, LC3 microtubule-associated protein light chain 3, ULK1 unc-51-like autophagy-activating kinase 1, Vec vector. All data are expressed as the mean ± SEM. ^*^P < 0.05, ^**^P < 0.01, ^***^P < 0.001

To investigate the role of tau in autophagosome formation, we first examined the expression level of LC3 in HEK293T cells (Fig. 1a), which had overexpression of hTau. To eliminate the influence of later events of autophagy by tau, we used 50 μmol/L vinblastine to block the fusion of autophagosome-lysosome. Overexpression of tau was first confirmed by Western blotting using Tau5, an antibody against total tau proteins. Simultaneously, the protein level of LC3 II was significantly decreased (*P* = 0.0073, Fig. 1a) with the decreased number of LC3 puncta (*P* < 0.0001, Fig. 1b) in the cells overexpressing tau measured respectively by Western blotting and immunofluorescence staining. By transmission electron microscopy, a decreased autophagosome formation was also detected in primary hippocampal neurons, which were infected with Lenti-eGFP-P2A-tau (Fig. 1c). Furthermore, overexpressing tau decreased the association of ULK1 and ATG13 (*P* = 0.0144), the main components of autophagosome formation, as measured by co-immunoprecipitation (Fig. 1d). These data suggest that overexpressing tau disrupts autophagosome formation.

### Overexpressing of tau upregulates mTORC1 activity

**Fig. 2:**
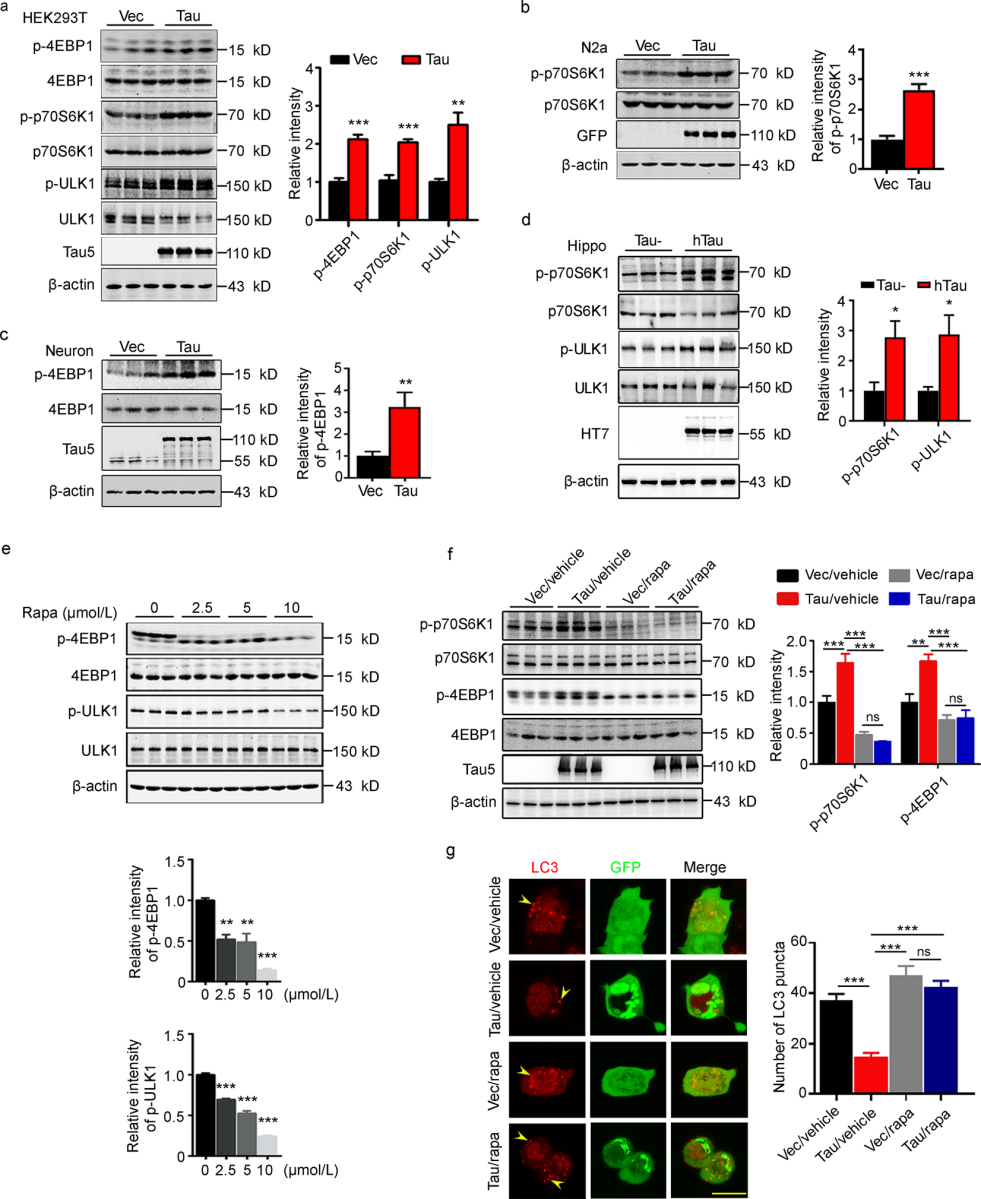
Overexpressing tau upregulates mTORC1 signaling. HEK293T (**a)** or N2a (**b)** cells transiently transfected with eGFP-tau, and the increased levels of phosphorylated 4EBP1, p70S6K1 and ULK1 were detected by Western blotting in the tau-OE group (*n* = 9). **c** The increased level of p-4EBP1 in primary cultured hippocampal neurons were detected by Western blotting in the tau-OE group (*n* = 9). **d** The hyperphosphorylation levels of p70S6K1 and ULK1 in the hippocampus of 12 month-old hTau mice were detected by Western blotting (*n* = 9). **e** HEK293T cells were treated with different concentrations of rapamycin for 6 h, and a dose-dependent reduction in p-4EBP1 and p-ULK1 was detected by Western blotting (*n* = 9). **f** Rapamycin decreased tau induced hyperphosphorylation of p70S6K1 and 4EBP1 in HEK293T cells, which was detected by Western blotting (*n* = 9). **g** Immunofluorescence staining results showed that the number of LC3 puncta (at least 22 cells were analyzed for each group) in HEK293T cells was recovered in tau/rapamycin group, scale bar = 20 μm. The yellow arrow points to the LC3 puncta. 4EBP1 eukaryotic translation initiation factor 4E-binding protein 1, GFP green fluorescent protein, Hippo hippocampus, LC3 microtubule-associated protein light chain 3, ns non-significant, p70S6K1 70 kDa ribosomal protein S6 kinase 1, Rapa rapamycin, ULK1 unc-51-like autophagy-activating kinase 1, Vec vector. All data are expressed as the mean ± SEM. ^*^P < 0.05, ^**^P < 0.01, ^***^P < 0.001

mTORC1 is involved in the most important upstream inhibitory pathway of autophagy. To explore how tau accumulation may affect the formation of autophagosomes, we transiently expressed tau in HEK293T and N2a cells, and then measured the total and the phosphorylated levels of 4EBP1, p70S6K1, and ULK1 by Western blotting. We found that transient overexpression of tau significantly increased the levels of phosphorylated 4EBP1 (*P* < 0.0001), p70S6K1 (*P* = 0.0001) and ULK1 (*P* = 0.0015) in HEK293T cells (Fig. 2a) and p70S6K1 (*P* < 0.0001) in N2a cells (Fig. 2b). In primary cultured hippocampal neurons, overexpressing tau also significantly increased the level of phosphorylated 4EBP1 (*P* = 0.0098) compared to the vector group (Fig. 2c). Compared with endogenous tau knockout mice, significantly increased levels of phosphorylated p70S6K1 (*P* = 0.0428) and ULK1 (*P* = 0.0467) were detected in the hippocampus of 12 month-old hTau transgenic mice (Fig. 2d). These results indicate that overexpressing tau inhibits autophagy via a mechanism involving mTORC1 signaling.

To further verify the role of mTORC1 in tau-induced deficits in autophagosome formation, we treated HEK293T cells with different concentrations of rapamycin, an inhibitor of mTORC1, and then measured the levels of phosphorylated 4EBP1 and ULK1. We found that simultaneous application of rapamycin at 2.5 μmol/L is already significantly decreased the phosphorylation level of 4EBP1 (*P* = 0.0026) and ULK1 (*P* < 0.0001, Fig. 2e). We also observed that rapamycin attenuated tau-induced upregulation of phosphorylated p70S6K1 (Vec/vehicle group vs. Tau/vehicle group, *P* = 0.0003; Vec/rapa group vs. Tau/rapa group, *P* = 0.1575; Tau/vehicle group vs. Tau/rapa group, *P* < 0.0001) and 4EBP1 (Vec/vehicle group vs. Tau/vehicle group, *P* = 0.0022; Vec/rapa group vs Tau/rapa group, *P* = 0.9971; Tau/vehicle group vs. Tau/rapa group, *P* < 0.0001) levels (Fig. 2f). Meanwhile, with immunofluorescence staining, we found that simultaneous application of rapamycin recovered tau-induced LC3 puncta formation deficit (Vec/vehicle group vs Tau/vehicle group, *P* < 0.0001; Vec/rapa group vs Tau/rapa group, *P* = 0.5567; Tau/vehicle group vs. Tau/rapa group, *P* < 0.0001, Fig. 2g). These data confirm that tau accumulation inhibits autophagosome formation by activating the mTORC1 pathway.

### Overexpressing tau activates the amino acid pathway which is upstream of the mTORC1

**Fig. 3:**
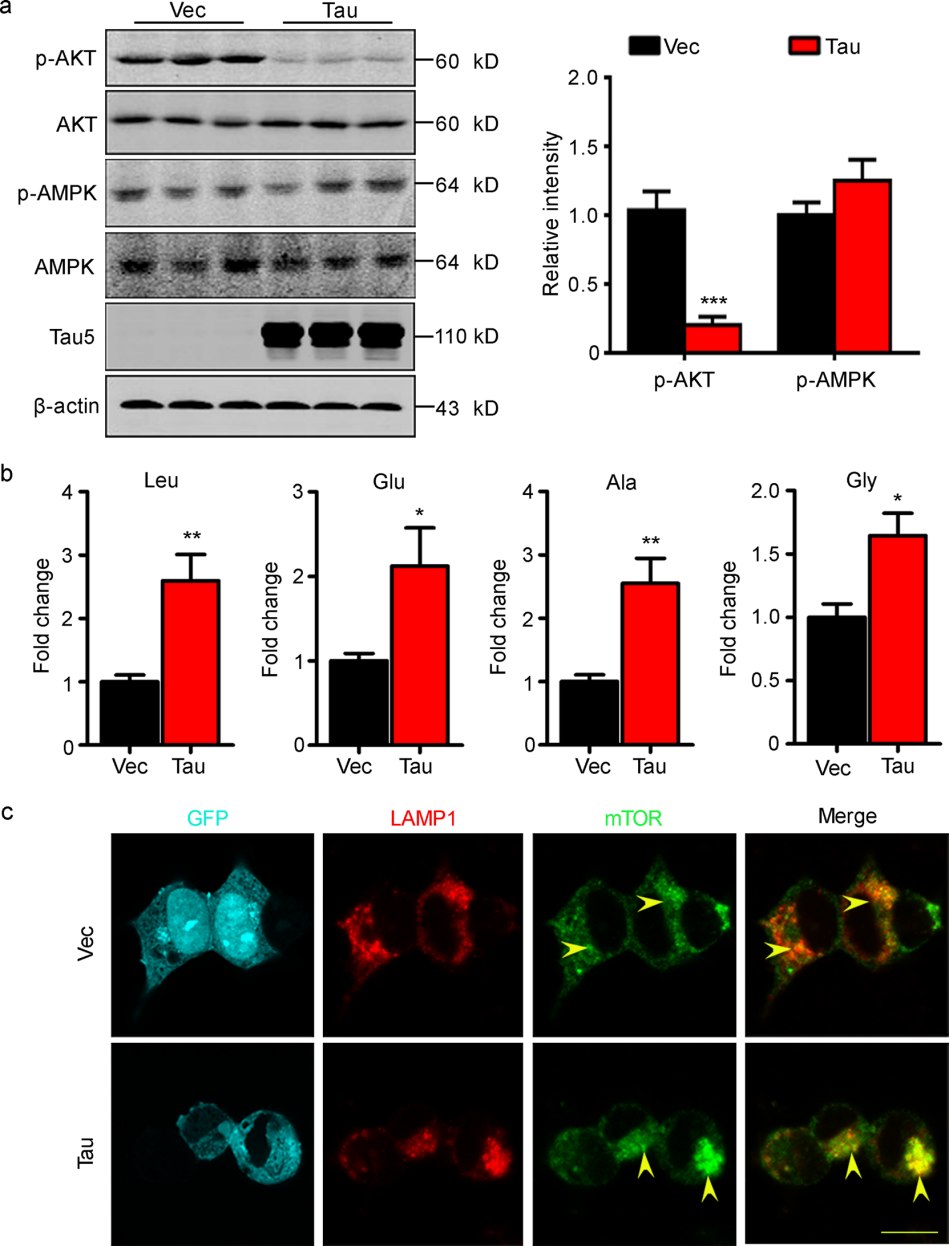
Overexpressing tau activates the amino acid pathway which is upstream of the mTORC1. **a** Total and phosphorylated AKT and AMPK in HEK293T cells were measured by Western blotting, and the results showed that the phosphorylated level of AKT was significantly decreased in the tau-OE group (*n* = 9). **b** Increased levels of intercellular leucine (Leu), glutamic acid (Glu), alanine (Ala), and glycine (Gly) in the tau-OE group were detected by HPLC (*n* = 6). **c** Increased colocalization of LAMP1 (Red) and mTOR (Green) in the tau-OE group of HEK293T cells was detected by immunofluorescence staining, scale bar = 20 μm. The yellow arrow points to colocalization of LAMP1 and mTOR. AKT RAC serine/threonine protein kinase, AMPK AMP-activated protein kinase, GFP green fluorescent protein, LAMP1 lysosome-associated membrane glycoprotein 1, mTOR mammalian target of rapamycin kinase, Vec vector. All data are expressed as the mean ± SEM. ^*^P < 0.05, ^**^P < 0.01, ^***^P < 0.001

To further explore the mechanisms underlying tau-induced mTORC1 activation, we measured the levels of the three upstream regulatory factors. We found that overexpressing tau inhibited AKT activity (*P* = 0.0002) and did not significantly influence AMPK activity (*P* = 0.1945) measured by Western blotting (Fig. 3a), which excluded these pathways as potential mediators of tau-induced mTORC1 activation. To explore the effect of the AA pathway, we measured the intracellular concentration of some essential amino acids by HPLC. The results showed that tau significantly increased the levels of leucine (*P* = 0.0038), glutamic acid (*P* = 0.0348), alanine (*P* = 0.0037), and glycine (*P* = 0.0104, Fig. 3b). By immunofluorescence staining, we observed increased cytoplasmic colocalization of LAMP1 and mTOR (Fig. 3c), which is a prerequisite for mTORC1 activation by amino acids. These data suggest that overexpressing tau may activate mTORC1 by upregulating the amino acid pathway.

### Inhibiting the amino acid pathway efficiently attenuates tau-induced mTORC1 activation

**Fig. 4:**
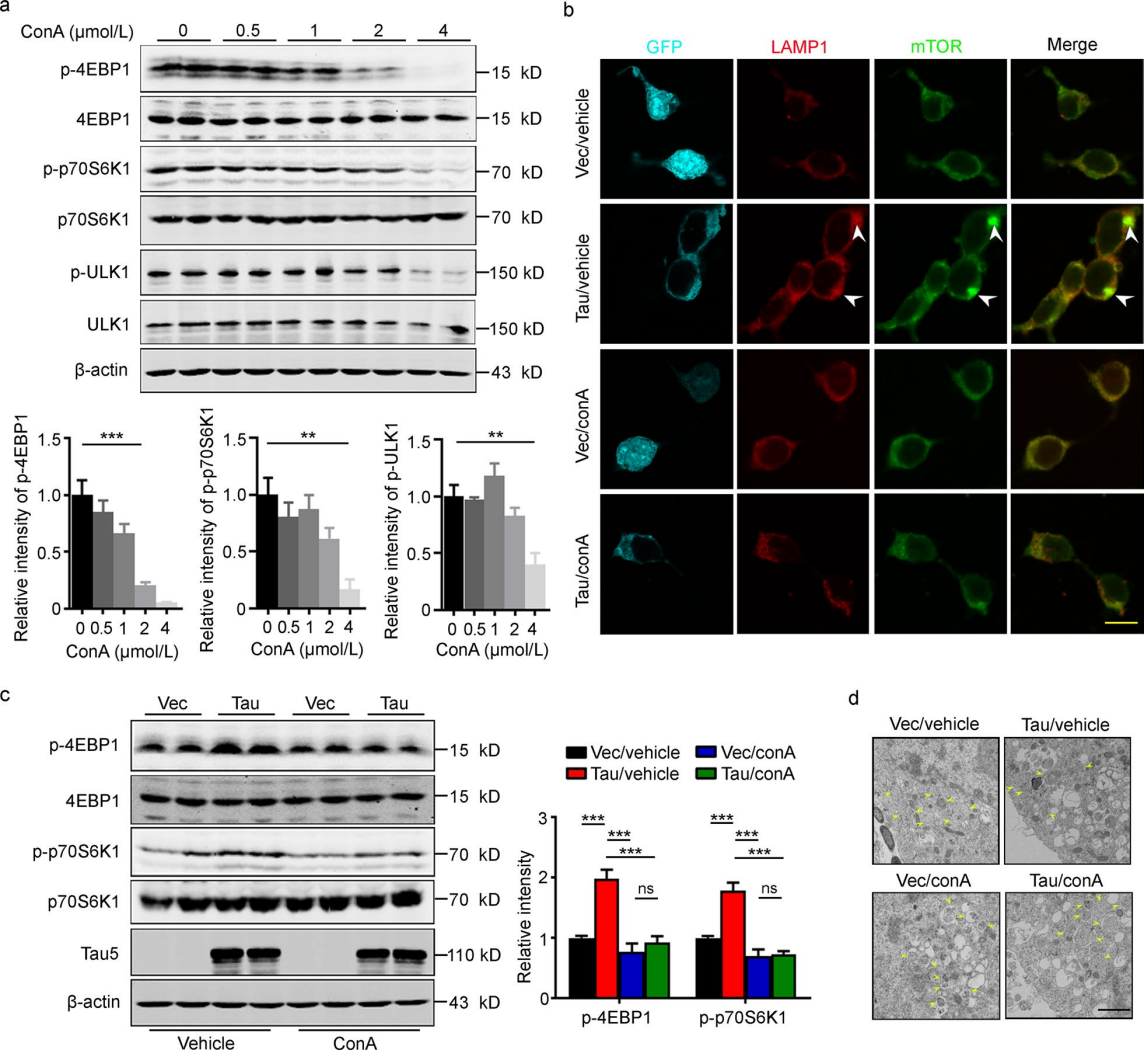
Inhibiting the amino acid pathway by ConA attenuates tau-induced mTORC1 upregulation. **a** Reduction in phosphorylated 4EBP1, p70S6K1 and ULK1 was detected by Western blotting in HEK293T cells, which were treated with different concentrations of concanamycin A (ConA, *n* = 6). **b** Application of ConA in HEK293T cells attenuated tau-induced mTORC1 upregulation, as evidenced by the decreased colocalization of LAMP1 (Red) and mTOR (Green), scale bar = 20 μm. The arrows point to colocalization of LAMP1 and mTOR. **c** Application of ConA in HEK293T cells attenuated tau-induced mTORC1 upregulation, as evidenced by the attenuated levels of p-4EBP1 and p-p70S6K1 (*n* = 6). **d** Application of ConA attenuated tau-induced impairment of autophagosomes formation in primary cultured hippocampal neurons, as imaged by transmission electron microscopy, scale bar = 1 μm. The yellow arrows point to the autophagosomes. 4EBP1 eukaryotic translation initiation factor 4E-binding protein 1, GFP green fluorescent protein, LAMP1 lysosome-associated membrane glycoprotein 1, mTOR the mammalian target of rapamycin kinase, ns non-significant, p70S6K1 70 kDa ribosomal protein S6 kinase 1, ULK1 unc-51-like autophagy-activating kinase 1, Vec vector. All data are expressed as the mean ± SEM. ^**^P < 0.01, ^***^P < 0.001

To further verify the role of the amino acid pathway in tau induced mTORC1 activation, we measured the levels of the phosphorylated 4EBP1, p70S6K1 and ULK1 in HEK293T cells, which were treated with different concentrations of concanamycin A (ConA), an inhibitor of the amino acid pathway. The results showed that ConA at 4 μmol/L significantly decreased the levels of phosphorylated 4EBP1, p70S6K1 and ULK1, while 1 μmol/L ConA did not affect the physiological activity of mTORC1 (Fig. 4a). Therefore, a concentration of 1 μmol/L ConA was used in subsequent experiments. ConA treatment remarkably attenuated tau-induced mTOR peri-lysosomal accumulation and mTORC1 upregulation, as measured by immunofluorescence staining (Fig. 4b) and detected the phosphorylation level of 4EBP1 (Vec/vehicle group vs. Tau/vehicle group, *P* = 0.0001; Vec/conA group vs. Tau/conA group, *P* = 0.7711; Tau/vehicle group vs. Tau/conA group, *P* < 0.0001) and p70S6K1 (Vec/vehicle group vs Tau/vehicle group, *P* = 0.0001; Vec/conA group vs Tau/conA group, *P* = 0.9955; Tau/vehicle group vs. Tau/conA group, *P* < 0.0001) by Western blotting (Fig. 4c), respectively. Furthermore, 1 μmol/L ConA significantly attenuated tau-induced disruption of autophagosome formation in cultured hippocampal neurons, as measured by electron microscopic imaging (Fig. 4d). These data further confirm that the increased AA signaling mediates tau-induced mTORC1 activation.

### N-terminal tau binds to TIA1-PRD and detains TIA1 in the cytoplasm to inhibit protein synthesis

**Fig. 5:**
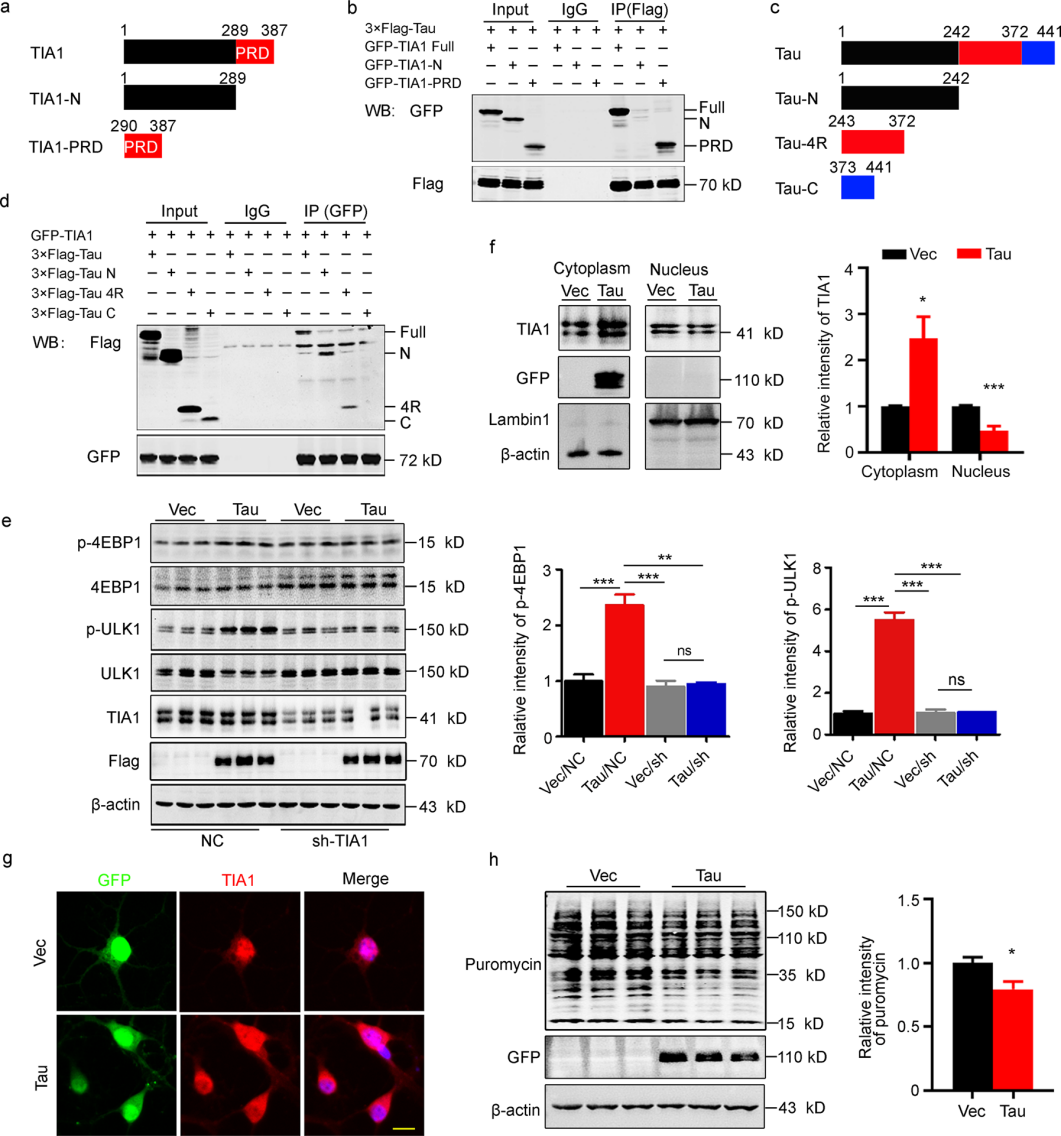
N-terminal tau binds to PRD-TIA1 to detain TIA1 in the cytoplasm and inhibit protein synthesis. **a** Schematic showing full-length TIA1, N-terminal TIA1 and the PRD of TIA1. **b** Tau could interact with TIA1 at its PRD domain as detected by coimmunoprecipitation in HEK293T cells. **c** Schematic showing constructs of full-length tau, N-terminal tau, four microtubule-binding repeats of tau, and C-terminal tau. **d** Tau-N and tau-4R but not Tau-C could efficiently bind TIA1, which was detected by coimmunoprecipitation in HEK293T cells. **e** N2a cells were cotransfected with 3 × Flag-tau and sh-TIA1 plasmids. The attenuated levels of p-4EBP1 and p-ULK1 in the tau/shTIA1 group were detected by Western blotting (*n* = 9). **f** N2a cells were transfected with eGFP-tau plasmids. The protein level of TIA1 was increased in the cytoplasm, while was decreased in the nucleus in the tau-OE group, as detected by Western blotting (*n* = 6). **g** Hippocampal neurons were infected with Lenti-eGFP-P2A-tau or Lenti-eGFP-P2A-C1, and the increased cytoplasmic localization of TIA1 (Red) was imaged by immunofluorescence staining (scale bar = 20 μm). **h** Decreased level of protein synthesis in N2a cells that transiently transfected with eGFP-tau plasmid was detected by Western blotting using an anti-puromycin antibody (*n* = 9). 4EBP1 the eukaryotic translation initiation factor 4E-binding protein 1, 4R four microtubule-binding repeats of tau, C C-terminal, GFP green fluorescent protein, IP immunoprecipitation, N N-terminal, NC normal control, ns non-significant, PRD prion-related domain, TIA1 T cell intracellular antigen 1, ULK1 unc-51-like autophagy-activating kinase 1, Vec vector. All data are expressed as the mean ± SEM. ^*^P < 0.05, ^**^P < 0.01, ^***^P < 0.001

To explore whether TIA1 is also involved in tau-induced upregulation of the AA pathway, we confirmed the interaction of tau with TIA1 and determined the specific region(s) of this interaction. First, we constructed eGFP-TIA1 Full (1–387, full length TIA1), eGFP-TIA1-N (1–289, N-terminal TIA1) and eGFP-TIA1-PRD (290–387, PRD-terminal TIA1) plasmids (Fig. 5a). The results of coimmunoprecipitation showed that TIA1-PRD could interact with tau (Fig. 5b). To identify the binding domain of tau with TIA1, we constructed plasmids of full-length tau and its fragments, including 3 × Flag-tau (1–441), 3 × Flag-tau-N (1–242), 3 × Flag-tau-4R (243–372) and 3 × Flag-tau-C (373–441, Fig. 5c). The results of coimmunoprecipitation showed that tau could bind TIA1 mainly with its N-terminal (Fig. 5d). These data suggest that tau may affect autophagosome formation through direct binding to the TIA1-PRD region.

To determine the role of TIA1 in tau-mediated activation of mTORC1, we constructed a TIA1 shRNA plasmid and found that knockdown of TIA1 remarkably inhibited tau-induced mTORC1 activation, which was represented by the attenuated phosphorylation of 4EBP1 (Vec/NC group vs. Tau/NC group, *P* = 0.0009; Vec/shTIA1 group vs. Tau/shTIA1 group, *P* = 0.9726; Tau/NC group vs. Tau/shTIA1 group, *P* = 0.0011) and ULK1 (Vec/NC group vs. Tau/NC group, *P* < 0.0001; Vec/shTIA1 group vs. Tau/shTIA1 group, *P* = 0.9980; Tau/NC group vs. Tau/shTIA1 group, *P* < 0.0001, Fig. 5e). TIA1 is primarily located in the nuclear compartment under physiological conditions, while tau is mainly expressed in the cytoplasm. Therefore, we studied whether overexpressing tau affected the cellular localization of TIA1. The results showed that overexpressing tau increased TIA1 protein level in the cytoplasm (*P* = 0.0109) with a decreased TIA1 level in the nucleus (*P* = 0.0009) compared with that of the vector group (Fig. 5f). Immunofluorescence staining was used to detect the localization of TIA1 in the hippocampal neurons, and found a higher cytoplasmic level of TIA1 in the tau-overexpressing neurons than that of the vector group (Fig. 5g). With the cytoplasmic detainment of TIA1, the level of newly synthesized protein significantly decreased (*P* = 0.0181, Fig. 5h). These results indicate that overexpressing tau may activate mTORC1 by detaining TIA1 in the cytoplasm, which also explains the increase in amino acid levels induced by tau.

### Blocking tau-TIA1 binding or promoting tau degradation attenuates tau-induced autophagy deficits

**Fig. 6:**
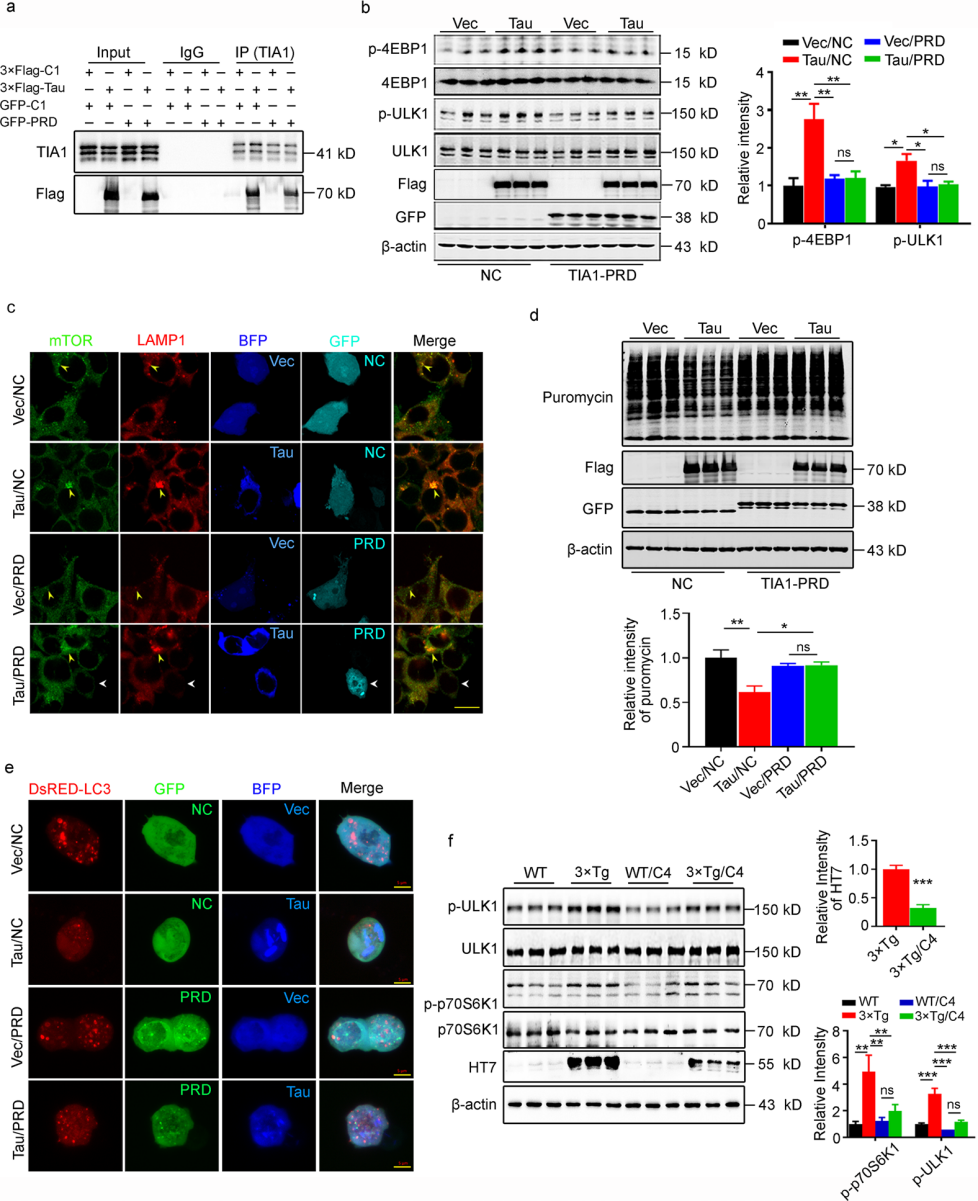
Expressing TIA1-PRD attenuates tau-induced autophagy dysfunction. **a** Expressing TIA1-PRD blocked tau and TIA1 binding in N2a cells detected by co-IP. **b** N2a cells were cotransfected with 3 × Flag-tau and GFP-PRD plasmids. Expressing TIA1-PRD attenuated tau-induced hyperphosphorylation of 4EBP1 and ULK1, which was detected by Western blotting (*n* = 9). **c** Expressing TIA1-PRD attenuated tau-induced perilysosomal aggregation of mTOR in HEK293T cells, which was imaged by immunofluorescence staining, scale bar = 20 μm. The yellow arrows point to the colocalization of LAMP1 and mTOR. **d** N2a cells were cotransfected with 3 × Flag-tau and GFP-PRD plasmids. Expressing TIA1-PRD recovered tau-induced dysfunction of protein synthesis, which was detected by Western blotting using anti-puromycin (*n* = 9). **e** Expressing TIA1-PRD recovered tau-induced dysfunction of autophagosome formation in HEK293T cells. The autophagosomes were imaged by immunofluorescence staining. scale bar = 5 μm. **f** 3 × Tg-AD mice were administered with PROTAC (C004019), and the protein level of human tau and the levels of phosphorylated p70S6K1 and ULK1 were detected by Western blotting (*n* = 9). 4EBP1 the eukaryotic translation initiation factor 4E-binding protein 1, BFP blue fluorescent protein, C4 C004019, GFP green fluorescent protein, IP immunoprecipitation, LAMP1 lysosome-associated membrane glycoprotein 1, LC3 microtubule-associated protein light chain 3, mTOR the mammalian target of rapamycin kinase, NC normal control, ns no significance, p70S6K1 70 kDa ribosomal protein S6 kinase 1, PRD prion-related domain, TIA1 T cell intracellular antigen 1, ULK1 unc-51-like autophagy-activating kinase 1, Vec vector, WT wild type. All data are expressed as the mean ± SEM. ^*^P < 0.05, ^**^P < 0.01, ^***^P < 0.001

To confirm the role of the tau-TIA1-PRD association in tau-induced dysfunction of autophagosome formation, we co-expressed TIA1-PRD and tau in N2a cells to block binding of tau with TIA1. We observed that overexpressing tau increased the interaction between tau and TIA1, and co-expressing TIA1-PRD remarkably decreased the tau-TIA1 interaction measured by coimmunoprecipitation (Fig. 6a). Co-expressing TIA1-PRD also abolished tau-induced hyperphosphorylation of 4EBP1 (Vec/NC group vs. Tau/NC group, *P* = 0.0043; Vec/PRD group vs. Tau/PRD group, *P* > 0.9999; Tau/NC group vs. Tau/PRD group, *P* = 0.0088) and ULK1 (Vec/NC group vs Tau/NC group, *P* = 0.0194; Vec/PRD group vs Tau/PRD group, *P* = 0.9885; Tau/NC group vs. Tau/PRD group, *P* = 0.0317, Fig. 6b). Furthermore, co-expressing TIA1-PRD with tau remarkably attenuated the tau-induced perilysosomal aggregation of mTOR measured by co-immunofluorescence staining with anti-LAMP1 and anti-mTOR antibodies (Fig. 6c), recovered protein synthesis inhibition measured by Western blotting using puromycin antibody (Vec/NC group vs. Tau/NC group, *P* = 0.0013; Vec/PRD group vs. Tau/PRD group, *P* = 0.9991, Tau/NC group vs. Tau/PRD group, *P* = 0.0111, Fig. 6d), and recovered autophagy dysfunction measured by immunofluorescence staining (Fig. 6e). In addition, hyperphosphorylation of p70S6K1 (*P* = 0.0015) and ULK1 (*P* = 0.0004) was detected in the hippocampus of 9.5 month-old 3 × Tg-AD mice (Fig. 6f). Subcutaneous administration of C004019 designed to simultaneously recruit tau and E3-ligase (Vhl) and thus selectively enhance ubiquitination and proteolysis of tau proteins, remarkably attenuated the hyperphosphorylation of p70S6K1 (*P* = 0.7108) and ULK1 (*P* = 0.2979) in the AD model mice (Fig. 6f). These data suggest that supplementation with TIA1-PRD can reverse the autophagy dysfunction induced by tau overexpression.

## Discussion

To date, there is a lack of effective therapy for AD. As increasing drug developments targeting β-amyloid clearance in AD have shown limited efficiency in halting disease progression in clinical trials, tau protein has attracted increasing attention as a new therapeutic target [30]. Accumulating research findings strongly suggest that tau accumulation plays a key role in AD neurodegeneration and cognitive deficits [31], and tau protein also mediates Aβ toxicity. Since tau accumulation and autophagy deficits are mutually exacerbated in AD [32], finding new therapeutic targets that modulate autophagy is important for attenuating or arresting pathological tau accumulation. Autophagy comprises several steps, and abnormalities at any step can induce autophagy deficits. We previously reported that human tau accumulation disrupts the fusion of autophagosomes and lysosomes by downregulating IST1, a component for ESCRT-III complex formation [19], which is the final step of autophagy. In the present study, we found that hTau accumulation could also inhibit mTORC1-associated autophagosome formation, which occurs early in the autophagy process. We also found that both inhibiting mTORC1 and downregulating tau protein expression could attenuate hTau-induced autophagy deficits. These findings provide new evidence supporting the use of these proteins as potential targets for AD drug development.

Intracellular accumulation of tau plays a crucial role in the progression of AD neurodegeneration and cognitive impairment, and autophagy deficit contributes to tau accumulation underlying AD [14–16]. Based on the chronic and progressive nature of AD pathogenesis and the discovery of the new cell viability-regulating functions of tau proteins [33, 34], we speculate that the accumulated tau may in turn affect the autophagy process and thus exacerbate tau aggregation. Indeed, we found in a recent study that increasing intracellular tau disrupted autophagosome-lysosome fusion with a mechanism involving reduced IST1-related ESCRT-III complex formation [19]. At the same time, we observed significantly reduced protein synthesis and reduced LC3 II level by tau, which could not be explained by the autophagosome-lysosome fusion deficit. In the present study, we further investigated how tau accumulation may affect protein synthesis. We found that intracellular tau accumulation could disrupt autophagosome formation by activating the TIA1-AA-mTORC1 pathway, in which decreased protein synthesis contributes to increased AA and mTORC1 activation.

The mTORC1 pathway is the most important inhibitory upstream pathway of autophagy. When the levels of amino acids or cellular energy are sufficient, mTORC1 is activated and thus induces phosphorylation of ULK1 and ATG13, the downstream autophagy initiation-related substrates of mTORC1, to impede their interaction and thus inhibit autophagosome formation [21, 35, 36]. We found that overexpressing tau decreased the protein expression level of LC3 II with a reduced number of LC3 puncta, and increased mTORC1 activation. Tau also inhibited the association of ULK1 and ATG13, and this ULK1-ATG13 complex is the main partner of ULK/ATG13/FIP200 autophagy-related complexes involved in autophagosome formation [37, 38]. To rule out the effect of tau on autophagosome-lysosome fusion as a mediator of this process, we administered vinblastine, a specific inhibitor of autophagosome-lysosome fusion [39, 40]. We also found that the application of the mTORC1 inhibitor rapamycin could remarkably attenuate tau-induced deficits in autophagosome formation. These data indicate that tau may inhibit autophagy by activating the mTORC1 pathway. Consistent with our results, several studies have also shown that mTORC1 is abnormally activated in the brains of AD patients during chronic disease progression [41–43], and inhibiting mTORC1 activity through genetic or pharmacologic manipulations can improve cognitive impairment in AD transgenic mice and prolong lifespan [25, 44, 45].

Then, we investigated how tau accumulation affects mTORC1 activity. From the published literature, no evidence suggests that tau can directly interact with mTORC1 components, including mTOR, Deptor, Raptor, the 40 kDa proline-rich AKT substrate (PRAS40) and the mammalian lethal with SEC13 protein 8 (mLST8) [46–48]. Therefore, we focused on the upstream regulatory pathways of mTORC1 activity. It is well known that the activity of mTORC1 is mainly regulated by three pathways, i.e., the AA pathway, AMPK pathway and phosphatidylinositol-3-kinase/AKT (PI3K/AKT) pathway. We found that overexpressing tau protein did not affect the phosphorylation level of AMPK, and the phosphorylation level of AKT at Ser473 was even decreased, which excluded the role of the AMPK and PI3K/AKT pathways as mediators of mTORC1 activation. Previous studies showed that stimulating starved cells with leucine or glutamine could activate mTORC1 and inhibit autophagy [49, 50]. The increased levels of amino acids could activate Rag guanosine triphosphatases (GTPases), which promote translocation of mTORC1 to the lysosomal surface, the site of mTORC1 activation [51, 52]. A recent study demonstrated a decreased protein synthesis in neurons of two transgenic mouse strains, K3 mice expressing K369I mutant tau and rTg4510 mice expressing P301L mutant tau [53]. The level of protein synthesis occurring in a cell is important for regulating the intracellular AA level. We found that overexpressing tau increased the intracellular concentrations of leucine, glutamic acid, alanine, and glycine, which could result from inhibited protein synthesis. Our data also showed that overexpressing tau increased the accumulation of mTOR in the peripheral region of the lysosome. Previous studies have shown that vacuolar H^+^-ATPase (v-ATPase) is required for amino acids to activate mTORC1, as it is engaged in extensive amino acid-sensitive interactions with Ragulator, a scaffolding complex that anchors the Rag GTPases to lysosomes [50]. In addition, in the presence of the v-ATPase inhibitor ConA, tau-induced mTORC1 activation was fully reversed. These results demonstrated that tau activated the amino acid pathway by increasing the concentration of intracellular amino acids and finally activating mTORC1. Nonetheless, other potential regulatory pathways may also exist, which deserve attention in future studies.

Previous studies have shown that aggregated tau can directly bind to the RNA-binding protein TIA1 to form larger RNA granules (RGs) and stress granules (SGs) [54]. To explore how tau accumulation may increase intercellular amino acid levels, we studied the role of the tau and TIA1 interaction. We constructed plasmids of tau fragments and TIA1 fragments. By transient co-transfecting and coimmunoprecipitation assays, we determined that tau could bind TIA1 by its N-terminal and 4R fragments, while the strongest binding site for TIA1 to tau was at its PRD-terminal. Overexpression of tau can increase TIA1 in the cytoplasm where it forms SGs. In support of our results, studies have also shown that the 290–387 fragment of TIA1 contains PRD, which can form prion-like aggregates and inhibit stress granules formation [54–56]. We also found that co-overexpressing TIA1-PRD and tau could attenuate tau-induced mTORC1 activation. The determination of the binding region provides the possibility to design drugs for the competitive inhibition of the tau-TIA1 interaction and thus to arrest tau-induced autophagy deficits.

**Fig. 7:**
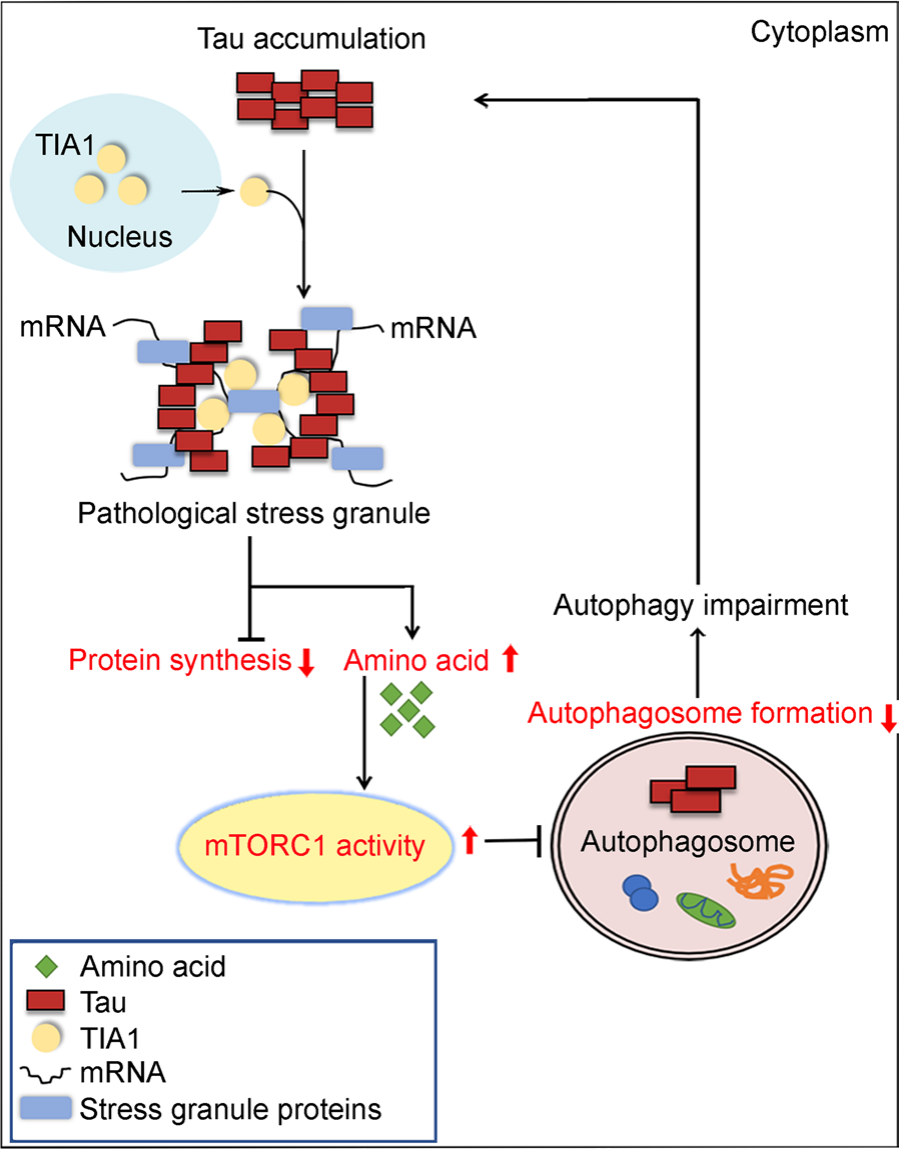
Schematic diagram showing the mechanisms of tau-induced autophagy dysfunction. The molecular mechanisms involve a direct binding of N-terminal tau to the TIA1-PRD to detain TIA1 in the cytoplasm, by which TIA1 is detained in the cytoplasm to form SGs, leading to the inhibition of protein synthesis, increases of intracellular AA concentration, mTORC1 activation, autophagosome reduction and finally autophagy deficit. The autophagy deficit in turn aggravates tau accumulation during chronic neurodegeneration, as observed in AD and related tauopathies. AA amino acid, AD Alzheimer’s disease, mTORC1 the mammalian target of rapamycin kinase complex 1, SGs stress granules, TIA1 T cell intracellular antigen 1, PRD the prion-related domain

Together, we found in the present study that the AD-like tau accumulation could inhibit autophagosome formation which is a critical step in the initiating states of the macroautophagy process. The molecular mechanisms involve a direct binding of N-terminal tau to the TIA1-PRD, formation of SGs, inhibition of protein synthesis, increases of intracellular AA, mTORC1 activation, autophagosome reduction and finally autophagy deficit. The autophagy deficit in turn aggravates tau accumulation during the course of chronic neurodegeneration, which is consistent with AD and related tauopathies (Fig. 7).

The role and molecular mechanism of SGs/RGs dysregulation in neurodegenerative diseases such as AD is a new and important research hotspot. However, it should be noted that the mRNA captured by the Tau-TIA1-related SGs has not yet been elucidated in this paper. We speculate that the Tau-TIA1 related SGs may prefer certain mRNAs depending on the types of other RNA-binding proteins in the Tau-TIA1 related SGs, which deserves further study.

## Conclusions

In general, the present study uncovered a new mechanism whereby tau accumulation inhibits autophagosome formation by activating TIA1-amino acid-mTORC1 signaling in AD. This work provides new insight into the mechanism of autophagy dysfunction in AD and provides a novel target for AD treatments.

## Data Availability

The datasets used and/or analysed during the current study are available from the corresponding author on reasonable request.

## Abbreviations

4EBP1: Eukaryotic translation initiation factor 4E-binding protein 1
4R: Four microtubule-binding repeats of tau
AA: Amino acid
AD: Alzheimer’s disease
AKT: RAC serine/threonine protein kinase
Ala: Alanine
AMPK: AMP-activated protein kinase
ANP32A: Acidic nuclear phosphoprotein 32 family member A
APP: Amyloid precursor protein
ATG13: Autophagy protein 13
ATG5: Autophagy protein 5
Aβ: β-Amyloid
BFP: Blue fluorescent protein
C: C-terminal
C4: C004019
Co-IP: Coimmunoprecipitation
ConA: Concanamycin A
ESCRT-III: Endosomal sorting complex required for transport III
FBS: Fetal bovine serum
FIP200: The FAK family kinase-interacting protein of 200 kDa
GFP: Green fluorescent protein
Glu: Glutamic acid
Gly: Glycine
Hippo: Hippocampus
HPLC: High performance liquid chromatography
HP-β-CD: 2-Hydroxylpropyl-beta-cyclodextrin
INHAT: Inhibitor of histone acetyl transferase
IP: Immunoprecipitation
IST1: Increased sodium tolerance 1
LAMP1: Lysosome-associated membrane glycoprotein 1
LC3: Microtubule-associated protein light chain 3
Leu: Leucine
mLST8: Mammalian lethal with SEC13 protein 8
mTOR: Mammalian target of rapamycin kinase
mTORC1: Mammalian target of rapamycin kinase complex 1
mTORC2: Mammalian target of rapamycin kinase complex 2
N: N-terminal
NC: Normal control
NFTs: Neurofibrillary tangles
ns: Non-significant
p70S6K1: 70 KDa ribosomal protein S6 kinase 1
PI3K: Phosphatidylinositol-3-kinase
PRAS40: 40 KDa proline-rich Akt substrate
PRD: Prion-related domain
Rapa: Rapamycin
RGs: RNA granules
SGs: Stress granules
TIA1: T cell intracellular antigen 1
ULK1: Unc-51-like autophagy-activating kinase 1
v-ATPase: The vacuolar H^+^-ATPase
Vec: Vector
WT: Wild type
